# Mechanisms for coping with submergence and waterlogging in rice

**DOI:** 10.1186/1939-8433-5-2

**Published:** 2012-02-27

**Authors:** Shunsaku Nishiuchi, Takaki Yamauchi, Hirokazu Takahashi, Lukasz Kotula, Mikio Nakazono

**Affiliations:** 1 Laboratory of Plant Genetics and Breeding, Graduate School of Bioagricultural Sciences, Nagoya University, Furo-cho, Chikusa, Nagoya 464-8601, Japan

**Keywords:** Aerenchyma, Barrier to radial O_2 _loss, Leaf gas films, Rice, Submergence, Waterlogging

## Abstract

Rice (*Oryza sativa *L.), unlike other cereals, can grow well in paddy fields and is highly tolerant of excess water stress, from either submergence (in which part or all of the plant is under water) or waterlogging (in which excess water in soil limits gas diffusion). Rice handles submergence stress by internal aeration and growth controls. A quiescence strategy based on *Submergence-1A *(*SUB1A*) or an escape strategy based on *SNORKEL1 *(*SK1*) and *SNORKEL2 *(*SK2*) is used for the growth controls. On the other hand, rice handles waterlogging stress by forming lysigenous aerenchyma and a barrier to radial O_2 _loss (ROL) in roots in order to supply O_2 _to the root tip. In this article, we summarize recent advances in understanding the mechanisms of responding to excess water stresses (*i.e*., submergence and waterlogging) in rice and other gramineous plants.

## Introduction

**Figure 1:**
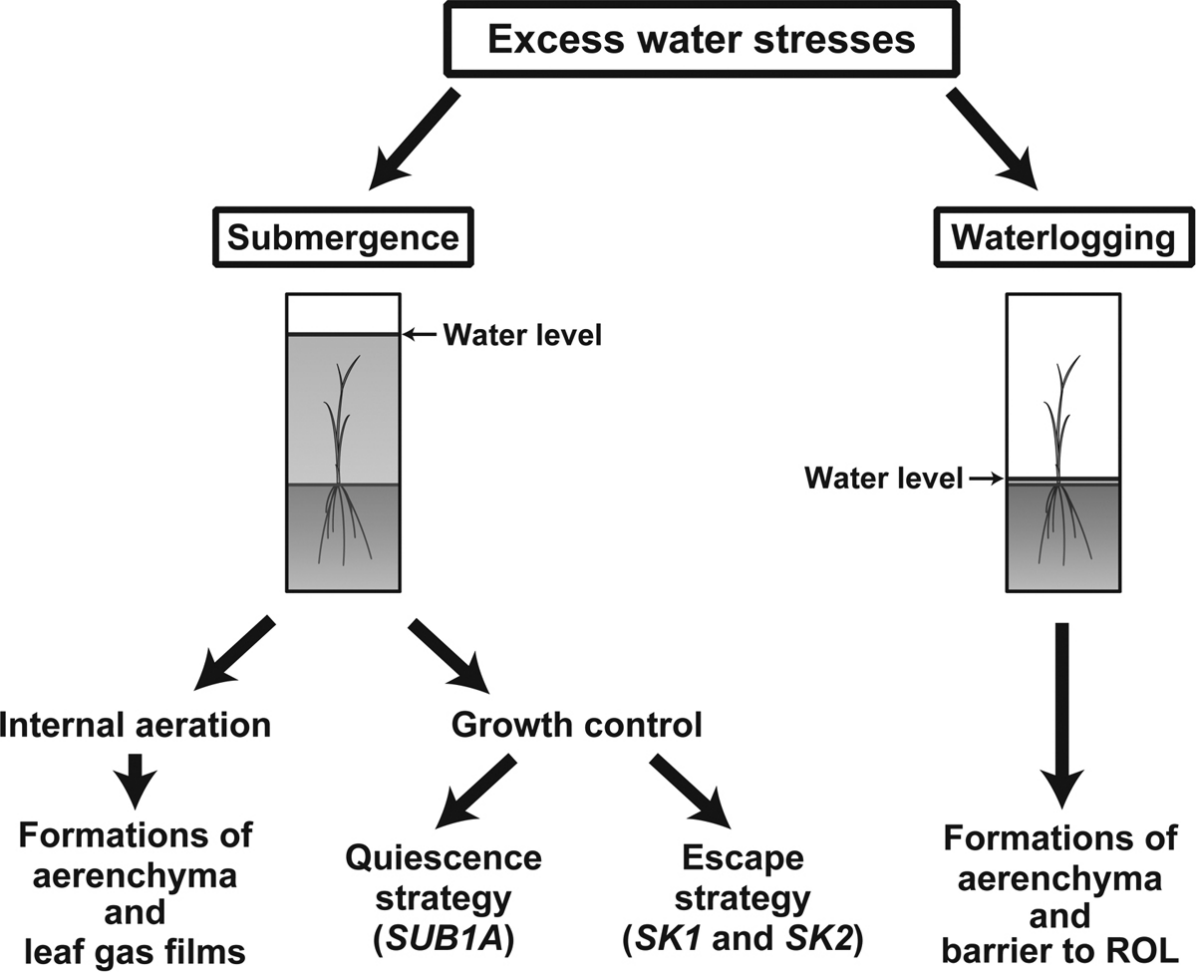
**Strategies of adaptation to excess water stresses in the form of submergence or waterlogging in rice plants**. Rice can adapt to submergence by internal aeration and growth control. For internal aeration, rice develops longitudinally forming aerenchyma and leaf gas films. On the other hand, some rice cultivars can survive under submergence by using special strategies of growth control: a quiescence strategy or an escape strategy. The *Submergence-1A *(*SUB1A*) gene is responsible for the quiescence strategy, which is important for survival under flash-flood conditions. The *SNORKEL1 *(*SK1*) and *SNORKEL2 *(*SK2*) genes are responsible for the escape strategy, which is important for survival under deepwater-flood conditions. Rice can adapt to soil waterlogging by forming aerenchyma and a barrier to radial O_2 _loss (ROL) in the roots.

Plants require water for growth but excess water that occurs during submergence or waterlogging is harmful or even lethal. A submerged plant is defined as "a plant standing in water with at least part of the terminal above the water or completely covered with water" (Figure 1; Catling 1992). Submergence subjects plants to the stresses of low light, limited gas diffusion, effusion of soil nutrients, mechanical damage, and increased susceptibility to pests and diseases (Greenway and Setter 1996; Ram et al. 1999). Basically, flooding (*i.e*., submergence) can be classified into "flash flooding" and "deepwater flooding" in accordance with the duration of flooding and the water depth (Bailey-Serres et al. 2010; Catling 1992; Jackson and Ram 2003). Flash flooding, which generally lasts less than a few weeks, is caused by heavy rain but the depth is not very deep. On the other hand, deepwater flooding, which lasts for several months, occurs during the rainy season, and the water depth reaches several meters (Catling 1992; Hattori et al. 2011).

Waterlogging is defined as a condition of the soil in which excess water limits gas diffusion (Figure 1; Setter and Waters 2003). Oxygen diffusivity in water is approximately 10,000 times slower than in air, and the flux of O_2 _into soils is approximately 320,000 times less when the soil pores are filled with water than when they are filled with gas (Armstrong and Drew 2002, Colmer and Flowers 2008). The principal cause of damage to plants grown in waterlogged soil is inadequate supply of oxygen to the submerged tissues as a result of slow diffusion of gases in water and rapid consumption of O_2 _by soil microorganisms. Oxygen deficiency in waterlogged soil occurs within a few hours under some conditions. In addition to the O_2 _deficiency, production of toxic substances such as Fe^2+^, Mn^2+^, and H_2_S by reduction of redox potential causes severe damage to plants under waterlogged conditions (Drew and Lynch 1980; Setter et al. 2009). Thus, growth and development of most plants, except for rice (*Oryza sativa *L.) and other wetland species, are impeded under waterlogged conditions.

Unlike other crop plants, rice has some adaptive traits for tolerance of submergence. One of the traits is formation of the longitudinal interconnection of gas spaces, called aerenchyma, that enables internal aeration between shoot and roots (Armstrong 1979; Colmer 2003a; Colmer and Pedersen 2008a) Moreover, leaf gas films, which are a micro-layer of air trapped between submerged leaves and the surrounding water, contribute to the internal aeration during submergence, thereby increasing submergence tolerance in rice (Colmer and Pedersen 2008b; Pedersen et al. 2009; Raskin and Kende 1983).

Many lowland rice cultivars, despite having an ability of internal aeration, are still sensitive to complete submergence. Their leaves and stems moderately elongate under complete submergence to reach the air-water interface, but their elongation growth can exhaust energy reserves and cause death when the flooding depth is deep and the flooding period is long (Bailey-Serres et al. 2010; Jackson and Ram 2003). However, some cultivars use two distinct strategies of growth controls to survive under submerged conditions. One of the strategies is a quiescence strategy [*i.e*., the low-oxygen quiescence syndrome (Colmer and Voesenek 2009)] (Figure 1), in which shoot elongation is suppressed to preserve carbohydrates for a long period (10-14 days) under flash-flood conditions. Submergence-tolerant cultivars can restart their growth during desubmergence by using preserved carbohydrates. Another strategy is an escape strategy [*i.e*., the low-oxygen escape syndrome (Bailey-Serres and Voesenek 2008; Colmer and Voesenek 2009)] (Figure 1), which involves fast elongation of internodes to rise above the water level and is used by deepwater rice cultivars. Both strategies depend on ethylene-responsive transcription factors (Hattori et al. 2009; Xu et al. 2006).

**Figure 2:**
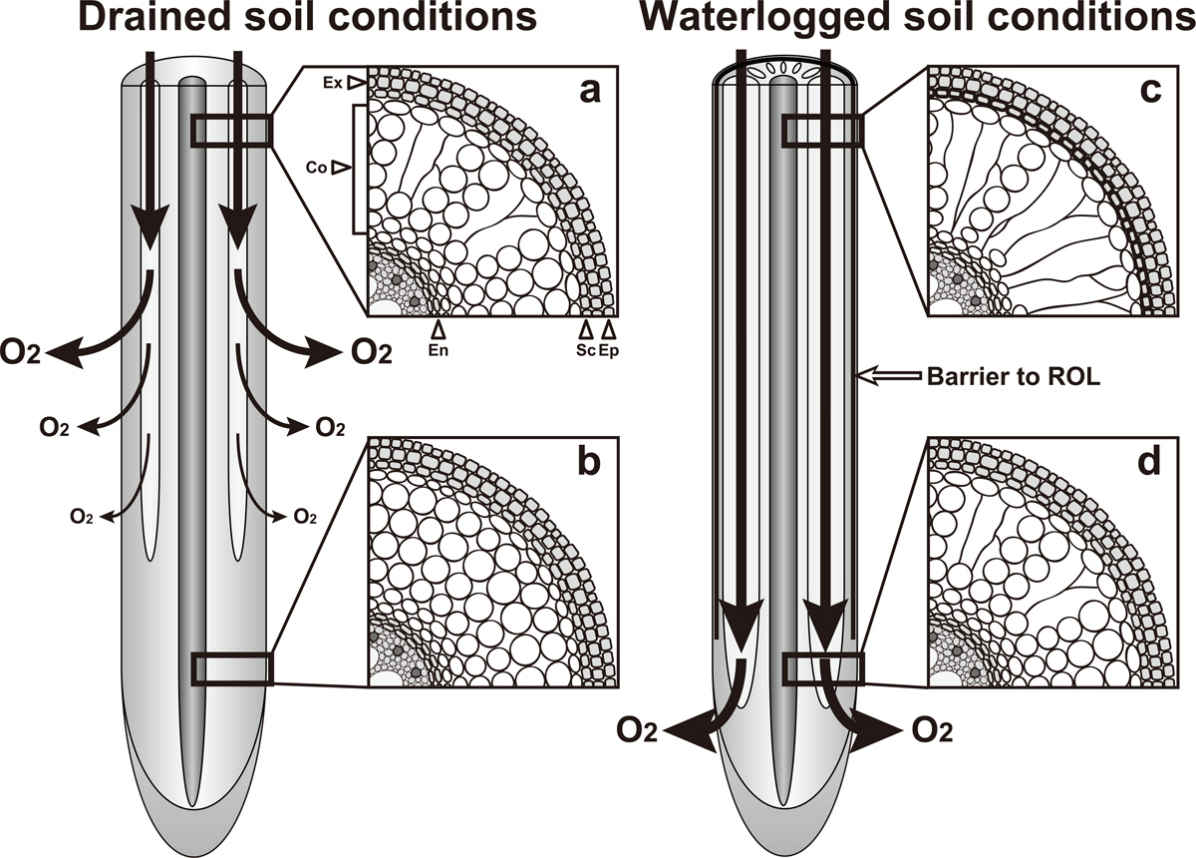
**Differences in lysigenous aerenchyma formation and patterns of radial O_2 _loss (ROL) in rice roots under drained soil conditions and waterlogged soil conditions**. Under drained soil conditions, lysigenous aerenchyma is constitutively formed, but a barrier to ROL is not formed; thus ROL at the basal part of the root decreases O_2 _diffusion to the apical part. By contrast, under waterlogged soil conditions lysigenous aerenchyma formation is enhanced and formation of the barrier to ROL is induced, resulting in the promotion of longitudinal O_2 _diffusion to the root apex. Under drained soil conditions, lysigenous aerenchyma is constitutively formed at the basal part of the roots (a), but it is not usually formed at the apical part of the roots (b). Under waterlogged soil conditions, lysigenous aerenchyma is induced at the basal part (c) and the apical part (d) of the roots. Lysigenous aerenchyma is more highly developed at the basal part of the roots (a, c) than at the apical part (b, d). Arrow thickness reflects the amount of O_2 _available. Ep, epidermis; Ex, exodermis; Sc, sclerenchyma; Co, cortex; En, endodermis.

The main adaptation of lowland rice to soil waterlogging is the formation of aerenchyma, which permits relatively unhindered transport of O_2 _from well-aerated shoots to submerged roots (Figures 1, 2; Armstrong 1979; Jackson and Armstrong 1999). Longitudinal diffusion of O_2 _towards the root apex can be further enhanced by induction of a barrier to radial O_2 _loss (ROL) that minimizes loss of O_2 _to the surrounding environment (Figures 1, 2). Furthermore, this barrier may impede the movement of soil-derived toxins (*i.e*., reduced metal ions) and gases (*e.g*. methane, CO_2_, and ethylene) into the roots (Armstrong 1979; Colmer 2003a; Greenway et al. 2006). Both upland and lowland rice species use these traits under waterlogged conditions (Colmer 2003b).

Some recent reviews summarized the mechanisms of flooding tolerance in plants (Bailey-Serres and Voesenek 2008; Bailey-Serres et al. 2010; Colmer and Voesenek 2009; Hattori et al. 2011; Nagai et al. 2010). In this review, we include recent discoveries that were not covered in the previous reviews and summarize what is known about the physiological and molecular mechanisms that contribute to tolerance to, or avoidance of, submergence (*i.e*., internal aeration and growth controls) and also adaptation to waterlogging (*i.e*., formations of aerenchyma and a barrier to ROL) in rice and other gramineous plants.

### Internal aeration in submerged plants

Effective internal aeration in plants is crucial to survive under submergence. In rice, aerenchyma is well developed in roots, internodes, sheaths, and the mid-rib of leaves (Colmer and Pedersen 2008a; Matsukura et al. 2000; Steffens et al. 2011) and contributes to the effective internal aeration between shoots and roots (Colmer 2003a; Colmer and Pedersen 2008a). Submerged leaves have gas films that aid O_2 _and CO_2 _exchange between leaves and the surrounding water, and thus increase underwater net photosynthesis by supplying CO_2 _during the day (under light conditions) and promote O_2 _uptake for respiration at night (Colmer and Pedersen 2008b; Pedersen et al. 2009; Raskin and Kende 1983). As a result, leaf gas films contribute to leaf sugar production by photosynthesis when under water, and in turn shoot and root dry mass (Pedersen et al. 2009). Removal of leaf gas films caused a decrease of O_2 _partial pressure (pO_2_) in roots when shoots were in darkness, suggesting that leaf gas films partially contribute to O_2 _transport from shoot to root (Pedersen et al. 2009; Winkel et al. 2011). Taken together, these findings indicate that leaf gas films are important for submergence tolerance in rice (Pedersen et al. 2009; Raskin and Kende 1983).

### Strategies of adaptation to flash-flood conditions

Catling (1992) defined submergence tolerance as "the ability of a rice plant to survive 10-14 days of complete submergence and renew its growth when the water subsides; there is no stem elongation during submergence." Under this definition, submergence tolerance indicates flash-flood tolerance. Generally, the seedlings of many lowland rice cultivars elongate their leaves to get oxygen at the water's surface under submerged conditions. However, because this shoot elongation requires large amounts of energy, most rice cultivars (*i.e*., flash-flood-intolerant cultivars) have poor ability to recover fully after the water recedes and eventually sustain severe damage or die (Jackson and Ram 2003). By contrast, the flash-flood-tolerant East Indian rice cultivar FR13A shows restricted shoot elongation and reduced energy consumption under submergence (Setter and Laureles 1996). The energy in FR13A plants is preserved during submergence, and upon desubmergence their growth can be restarted by using this energy (Fukao et al. 2006; Singh et al. 2001). There is therefore a negative correlation between shoot elongation and survival rate under complete submergence (Setter and Laureles 1996).

FR13A has the *Submergence-1 *(*SUB1*) locus on chromosome 9 (Xu and Mackill 1996). Xu et al. (2006) discovered that the *SUB1 *locus contains *SUB1A*, *SUB1B*, and *SUB1C*, all of which encode ethylene response factors and are upregulated under submergence, but only *SUB1A *is responsible for the flash-flood tolerance. The near-isogenic line M202(*SUB1*), which was generated by introgression of the *SUB1 *region from FR13A into the flash-flood-intolerant cultivar M202, shows restricted shoot elongation under submergence, as does FR13A (Bailey-Serres et al. 2010; Fukao et al. 2006; Xu et al. 2006). To preserve energy and carbohydrates, M202(*SUB1*) suppresses the expression of genes encoding α-amylase and sucrose synthase, which are involved in starch and sucrose metabolism (Fukao et al. 2006). In addition, *SUB1A *positively regulates the genes involved in alcohol fermentation and thus promotes acclimation of plants to flash-flood conditions (Fukao et al. 2006). Fukao and Bailey-Serres (2008) reported that *SUB1A *also enhances the expression of genes encoding SLENDER RICE-1 (SLR1) and SLR1 like 1 (SLRL1), which are key repressors of gibberellin (GA) signaling in rice; it also negatively regulates the GA response in order to restrict shoot elongation under submergence. Recently, it was shown that *SUB1A *expression is also induced by drought and oxidative stress upon desubmergence (Fukao et al. 2011). SUB1A positively regulates the expressions of genes involved in ABA-mediated acclimation to drought conditions. Moreover, under oxidative stress, SUB1A promotes the expression of genes related to the detoxification of reactive oxygen species (ROS) and reduces the accumulation of ROS. As a result, M202(*SUB1*) has a higher drought and oxidative tolerance than M202 (Fukao et al. 2011). In rain-fed rice fields, inadequate water management is prone to cause flooding and drought. Thus, as submergence and drought stresses can cause severe decreases in rice production in rain-fed rice fields, introgression of the *SUB1A *gene into rice cultivars intolerant of submergence and drought is a promising way of increasing rice productivity in these fields (Fukao et al. 2011).

### Strategies for adaptation to deepwater-flood conditions

Deepwater flooding lasts for several months, and O_2 _deficiency causes energy depletion in plants. To survive under deepwater-flood conditions, rice plants must escape from the flooding. A unique trait of deepwater rice is that its internodes rapidly and substantially elongate to avoid deepwater flooding. Remarkably, some deepwater rice cultivars can increase their height by 25 cm/day (Vergara et al. 1976). This rapid elongation allows the leaf tips to extend above the water surface and enables the rice plants to efficiently photosynthesize and exchange gases for respiration (Bailey-Serres and Voesenek 2008). During internode elongation, ethylene biosynthesis is activated and the accumulated ethylene regulates the increases in GA content and decrease in ABA content. As internode elongation is promoted by GA or repressed by ABA, the increased GA/ABA ratio contributes to the elongation (Kende et al. 1998; Sauter 2000). Indeed, GA activates the expression of cell division-related genes (Sauter et al. 1995; van der Knaap et al. 1997), and thus active cell division is observed at the intercalary meristem in the internode under submergence (Métraux and Kende 1984). Moreover, high levels of expression of genes encoding expansin, which is involved in cell-wall loosening (Cho and Kende 1997a, b, c; Lee and Kende 2001), and changes in the orientation of cellulose microfibrils are observed in the internode during internode elongation (Sauter et al. 1993). Aerenchyma formation occurs in the internodes simultaneously with their elongation, and is enhanced by ethylene (Steffens et al. 2011). Growth of adventitious roots, which is preceded by death of epidermal cells that cover the root primordia (Mergemann and Sauter 2000; Steffens and Sauter 2005), is also promoted by ethylene (Steffens et al. 2006).

Recently, Hattori et al. (2009) identified the *SNORKEL1 *(*SK1*) and *SNORKEL2 *(*SK2*) genes responsible for internode elongation in deepwater rice. Non-deepwater rice (*i.e*., lowland rice) into which *SK1 *or *SK2 *had been introduced showed internode elongation in the same way as deepwater rice, indicating that the *SK *genes are key factors for the escape strategy of deepwater rice under deepwater-flood conditions. Because of space considerations, details of the function of *SK *genes have not been included in this review, but they have been summarized by Nagai et al. (2010) and Hattori et al. (2011).

### Strategies of adaptation to waterlogging: (1) aerenchyma formation

Formation of aerenchyma is essential to the survival and functioning of plants subjected to waterlogging. The aerenchyma contributes to O_2 _supply from shoots to roots and to the ventilation of gases (*e.g*. CO_2 _and methane) from roots to shoots (Colmer 2003a; Evans 2003). The ventilation of gases in aerenchyma is mainly caused by gas diffusion in rice, but in some wetland species with 'through-flow pathways' (*e.g*. along rhizomes), gas flows can also occur by humidity- and Venturi-induced pressure flows (*e.g*. *Phragmites australis*; Armstrong et al. 1996). The aerenchyma may provide a photosynthetic benefit by concentrating CO_2 _from root respiration and transporting it to the leaf intercellular spaces in some wetland plant species (Constable and Longstreth 1994).

In general, aerenchyma can be classified into two types: (i) schizogenous aerenchyma, which develops by cell separation and differential cell expansion that creates spaces between cells, in *e.g*. *Rumex palustris*; and (ii) lysigenous aerenchyma, formed by the death and subsequent lysis of some cells, *e.g*., in rice (Jackson et al 1985a), maize (Drew et al 1981), wheat (Trought and Drew 1980), and barley (Arikado and Adachi 1955). In the roots, lysigenous aerenchyma forms in the cortex (Figure 2), whereas in the stems it can form in the cortex and pith cavity (Armstrong 1979).

In some wetland plant species such as rice, root lysigenous aerenchyma is constitutively formed under drained soil conditions (*i.e*., aerobic conditions; Jackson et al. 1985a), and its formation can be further enhanced during soil waterlogging (Figure 2; Colmer et al 2006; Justin and Armstrong 1991; Shiono et al. 2011; Visser and Bögemann 2006). In rice, aerenchyma formation is initiated at the apical parts of the roots and gradually expands to the basal parts of the roots (Figure 2; Ranathunge et al. 2003). Fully developed aerenchyma, which is observed on the basal parts of roots, separates the inner stele from the outer cell layers (*i.e*., sclerenchyma, hypodermis/exodermis, and epidermis) of the roots (Figure 2; Armstrong and Armstrong 1994; Kozela and Regan 2003; Ranathunge et al. 2003). Strands of remaining cells and cell walls separate gas spaces in the cortex, forming radial bridges, which are important for the structural integrity of the root and for both apoplastic and symplastic transport of nutrients (Figure 2; Drew and Fourcy 1986). During aerenchyma formation in rice root, cell death begins at the cells in the mid-cortex and then spreads out radially to the surrounding cortical cells (Kawai et al. 1998). The epidermis, hypodermis/exodermis, endodermis, and stele are unaffected, indicating that lysigenous aerenchyma formation occurs by closely controlled mechanisms (Yamauchi et al. 2011).

By contrast, in non-wetland plant species such as maize, wheat, and barley, root lysigenous aerenchyma does not form under well-drained soil conditions, but it may be induced by poor aeration (McDonald et al. 2001; McPherson 1939; Trought and Drew 1980). Generally, induction of aerenchyma formation takes 24-72 hours after the start of anaerobic treatment (Haque et al. 2010; Malik et al. 2003; Rajhi et al. 2011). In addition, aerenchyma formation is less extensive in non-wetland plant species than in wetland plant species (Armstrong 1979; Colmer and Voesenek 2009). Thus, non-wetland plants are less tolerant to waterlogging than wetland plants, such as rice.

#### Signaling of lysigenous aerenchyma formation

In rice and maize, ethylene is implicated in the induction of lysigenous aerenchyma formation (Drew et al. 2000; Jackson and Armstrong 1999; Justin and Armstrong 1991; Konings 1982; Shiono et al. 2008). Rice roots form lysigenous aerenchyma constitutively even under well-aerated conditions (Jackson et al. 1985a; Justin and Armstrong 1991; Shiono et al. 2011). Lysigenous aerenchyma formation in rice roots can be further increased by ethylene treatment under aerated conditions and decreased by treatment with an ethylene perception inhibitor (*e.g.* silver ions) under stagnant (0.1% agar) deoxygenated conditions (which mimics hypoxic/anoxic conditions in waterlogged soils; Wiengweera et al. 1997), although cultivars (*e.g.* Norin 36 and RB3) differ in their sensitivity to ethylene (Justin and Armstrong 1991). Justin and Armstrong (1991) also pointed out that consideration of the lengths of the roots sampled was important for comparison of aerenchyma formations between treatments. More recently, ethylene has been shown to increase root aerenchyma formation in another rice cultivar, Calrose (Colmer et al. 2006). In maize roots, ethylene biosynthesis is stimulated by enhancing the activity of 1-aminocyclopropene-1-carboxylic acid (ACC) synthase and ACC oxidase at the beginning of aerenchyma formation (He et al. 1996a). Thus, treatment of maize roots with inhibitors of ethylene biosynthesis (*e.g*. aminoethoxyvinylglycine, aminooxyacetic acid (AOA), and cobalt chloride) or action (*e.g*. silver ions) effectively blocks aerenchyma formation under hypoxic conditions (Drew et al. 1981; Jackson et al. 1985b; Konings 1982). These observations indicate that ethylene works as a trigger for the induction of aerenchyma formation in rice and maize. On the other hand, in the roots of another wetland species (*Juncus effusus*), lysigenous aerenchyma formation is not affected by treatment with ethylene or the ethylene perception inhibitor 1-methylcyclopropene (1-MCP; Visser and Bögemann 2006). To determine whether ethylene is (or is not) a common factor in regulation of the induction of lysigenous aerenchyma formation in the roots of wetland species, the effect of ethylene on aerenchyma formation should be studied by treatment of a wider range of wetland species while considering the possible influences of root length and tissue age along the root axes (Justin and Armstrong 1991; Visser and Bögemann 2006).

Ethylene-responsive lysigenous aerenchyma formation is affected by chemical inhibitors or stimulators of programmed cell death (PCD) and other signaling pathways. Heterotrimeric G-protein-, phospholipase C (PLC)-, inositol 1,4,5-trisphosphate (IP3)-, or Ca^2+^-dependent signaling pathways are involved in the process of lysigenous aerenchyma formation in maize roots (Drew et al. 2000; He et al. 1996b). It has been proposed that, under oxygen deprivation, Ca^2+ ^is released from mitochondria into the cytosol (Subbaiah et al. 1994); the elevated cytosolic Ca^2+ ^may provoke subsequent activation of kinases and phosphatases during aerenchyma formation (Subbaiah and Sachs 2003). Lysigenous aerenchyma formation is also induced by okadaic acid, an inhibitor of protein phosphatases, and is repressed by K252a, an inhibitor of protein kinases (Drew et al. 2000; He et al. 1996b). These calcium-dependent signalings may result in activation of expression of the genes responsible for aerenchyma formation (Drew et al. 2000; Subbaiah and Sachs 2003). PCD is a tightly regulated pathway that accompanies the activation of specific biochemical pathways (Greenberg 1996). PCD is distinguished from necrosis, which occurs by uncontrolled, accidental cell death without activation of signaling pathways (Drew et al. 2000; Gunawardena et al. 2001a). Cell death during lysigenous aerenchyma formation is similar to apoptosis in animal cells, which includes DNA fragmentation, nuclear condensation, and nuclear and plasma membrane blebbing (Drew et al. 2000).

One of the final steps in lysigenous aerenchyma formation is degradation of the cell wall, which is mediated by cell-wall modification or degradation enzymes. Changes in esterified and de-esterified pectins in the cell wall of the maize cortex are observed during cell death (Gunawardena et al. 2001b). Subsequently, the cell wall is degraded by the combined action of pectolytic, xylanolytic, and cellulosolytic enzymes (Evans 2003; Jackson and Armstrong 1999). The activity of cellulase (CEL) is increased by treatment with ethylene, okadaic acids, and reagents that increase intracellular Ca^2+ ^levels, whereas CEL activity is decreased by treatment with K252a and inhibitors of Ca^2+ ^increase (He et al. 1996b). In maize roots, expression of a gene encoding xyloglucan endotransglycosylase (XET) is induced by waterlogging, and its induction is inhibited by treatment with the ethylene biosynthesis inhibitor AOA (Saab and Sachs 1996). Treatment with AOA prevents the formation of lysigenous aerenchyma, suggesting that induction of XET production in response to ethylene is involved in aerenchyma formation through cell-wall modification (Saab and Sachs 1996).

On the basis of this evidence, Evans (2003) proposed that cell death in the root cortex during lysigenous aerenchyma formation can be classified into five steps: (1) perception of hypoxia and initiation of ethylene biosynthesis; (2) perception of ethylene signaling by cells of the mid-cortex; (3) initiation of cell death with loss of ions to the surrounding environment, plasma membrane invagination, and formation of small vesicles; (4) chromatin condensation, increased activity of cell-wall hydrolytic enzymes, and the surrounding of organelles by membranes; and (5) cell-wall degradation, cell lysis, and absorption of the cell contents and water by the surrounding cells.

#### Genes associated with lysigenous aerenchyma formation

So far, studies of lysigenous aerenchyma formation have been done mainly from a physiological perspective. However, the genes involved in lysigenous aerenchyma formation in the root have not been identified. Recently, Rajhi et al. (2011) identified genes associated with lysigenous aerenchyma formation in maize roots by using a microarray analysis combined with laser microdissection. They found that Ca^2+ ^signaling-related genes encoding Calcineurin B-like protein and Calmodulin-like protein were upregulated under waterlogged conditions, and their expression levels were higher in the cortical cells than in the stelar cells. Waterlogging also induces the expression of cell-wall modification-related genes (*e.g. XET *and *CEL*). Induction of the expressions of calcium signaling- and cell wall modification-related genes is suppressed by treatment with 1-MCP. These results support the previously proposed mechanism of ethylene-mediated lysigenous aerenchyma formation (Jackson and Armstrong 1999; Drew et al. 2000; Evans 2003).

**Figure 3:**
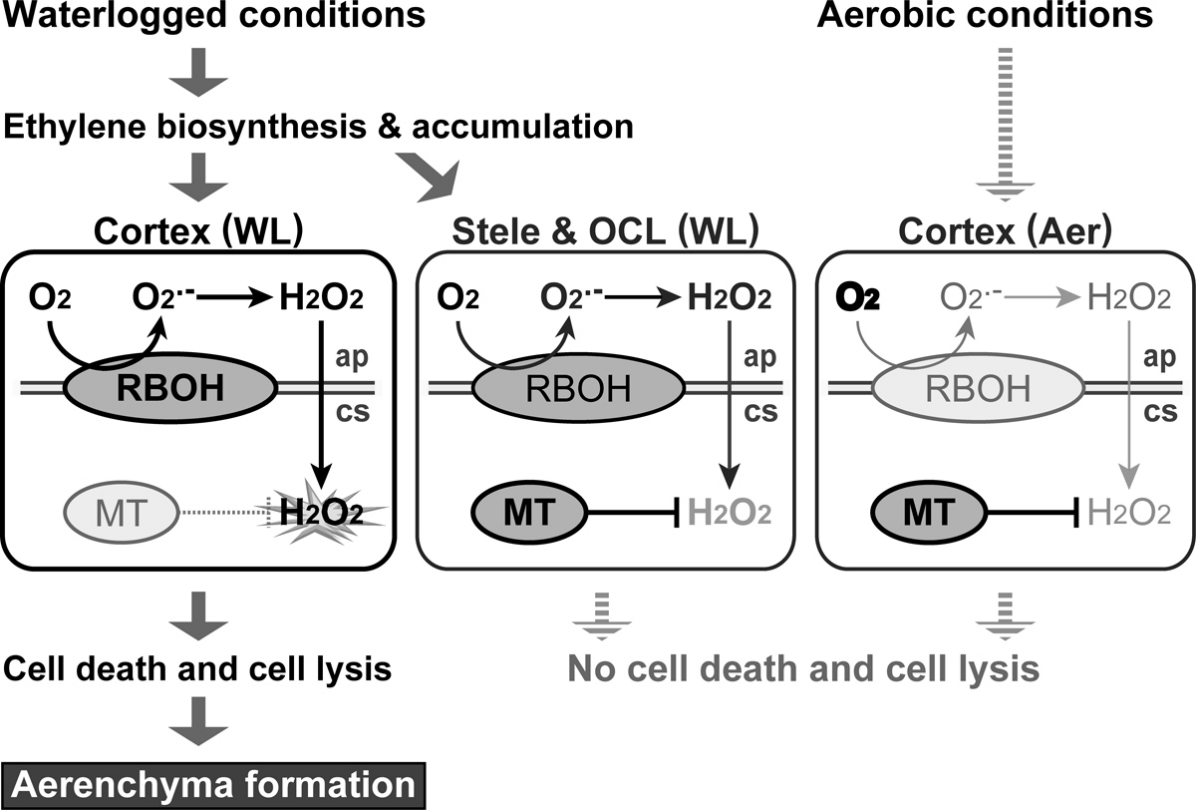
**Model of lysigenous aerenchyma formation**. Waterlogging promotes biosynthesis and accumulation of ethylene, followed by induction of *RBOH *expression. RBOH activity leads to production and accumulation of O_2_^·-^ at the apoplast. The O_2_^·-^ is spontaneously or enzymatically converted to H_2_O_2_, which can easily diffuse into the cytosol through the plasma membrane. Under waterlogged conditions, in the cytosol of stelar cells and cells in the outer cell layers, H_2_O_2 _and other ROS are scavenged by constitutively-expressed MT. By contrast, in the cortical cells, the decreased *MT *expression prevents ROS scavenging, thereby leading to greater ROS accumulation, which activates the subsequent processes of programmed cell death and lysis of the cortical cells (*i.e*., lysigenous aerenchyma formation). Under aerobic conditions, the *RBOH *gene is expressed at low level and the *MT *gene is constitutively expressed in the cortical cells. WL, waterlogged conditions; Aer, aerobic conditions; OCL, outer cell layers; ap, apoplast; cs, cytosol.

A gene encoding respiratory burst oxidase homolog (RBOH; a plant homolog of gp91^phox ^in mammalian NADPH oxidase), which has a role in ROS generation (Torres and Dangl 2005), is upregulated strongly in the cortical cells and slightly less strongly in the stelar cells and the outer cell layers of maize roots (Figure 3; Rajhi et al. 2011; Yamauchi et al. 2011). On the other hand, a gene encoding metallothionein (MT), which has a role in ROS scavenging (Wong et al. 2004, Xue et al. 2009), is constitutively expressed in all of the cortical cells, the stelar cells, and the outer cell layers of maize roots under aerobic conditions. By contrast, under waterlogged conditions the *MT *gene is hardly expressed at all in the cortical cells but is still highly expressed in the stelar cells and the outer cell layers (Figure 3; Rajhi et al. 2011; Yamauchi et al. 2011). These results suggest that H_2_O_2 _and other ROS are scavenged by the constitutively expressed MT in stelar cells and the outer cell layers, whereas in the cortical cells decreased *MT *expression prevents ROS scavenging, thereby leading to greater ROS accumulation, which activates the subsequent processes of PCD (*i.e*., lysigenous aerenchyma formation) in maize roots (Figure 3). Interestingly, upregulation of *RBOH *and downregulation of *MT *also occur in rice roots during inducible aerenchyma formation under anaerobic conditions (Yamauchi and Nakazono, unpublished). Similarly, ethylene-promoted downregulation of expression of a gene encoding MT2b enhances the accumulation of H_2_O_2 _produced by NADPH oxidase and thus induces epidermal cell death in rice (Steffens and Sauter 2009) or aerenchyma formation in rice internodes (Steffens et al. 2011). These results suggest that downregulation of *MT *genes plays an important role in tissue-specific or cell type-specific PCD in rice and maize.

### Strategies of adaptation to waterlogging: (2) formation of a barrier to ROL

Oxygen molecules diffusing longitudinally through aerenchyma toward the root tips may be either consumed by respiration or diffused radially to the rhizosphere (Armstrong 1979; Colmer 2003a). ROL, the flux of O_2 _from the aerenchyma to the soil, is determined by the concentration gradient, the physical resistance to O_2 _diffusion in a radial direction, and consumption of O_2 _by cells along this radial diffusion path (Armstrong 1979; Colmer 2003a). ROL aerates the rhizosphere and is therefore considered to be of adaptive significance in plants growing in waterlogged soil (Armstrong 1979; Blossfeld et al. 2011; Colmer 2003a; Neubauer et al. 2007). However, ROL reduces the supply of O_2 _to the root apex and thereby causes a decrease in root length in anaerobic soil (Armstrong 1979; Colmer 2003a; Colmer et al. 1998; Jackson and Armstrong 1999).

The roots of many wetland species, including rice, have the ability to prevent ROL to the rhizosphere by forming a barrier in the root peripheral cell layers exterior to the aerenchyma (Figure 2; McDonald et al. 2002; Visser et al. 2000). This adaptive trait enhances longitudinal O_2 _diffusion through the aerenchyma towards the root apex by diminishing losses of O_2 _to the rhizosphere, thereby enabling the roots to elongate into anaerobic substrates (Armstrong 1979).

**Figure 4:**
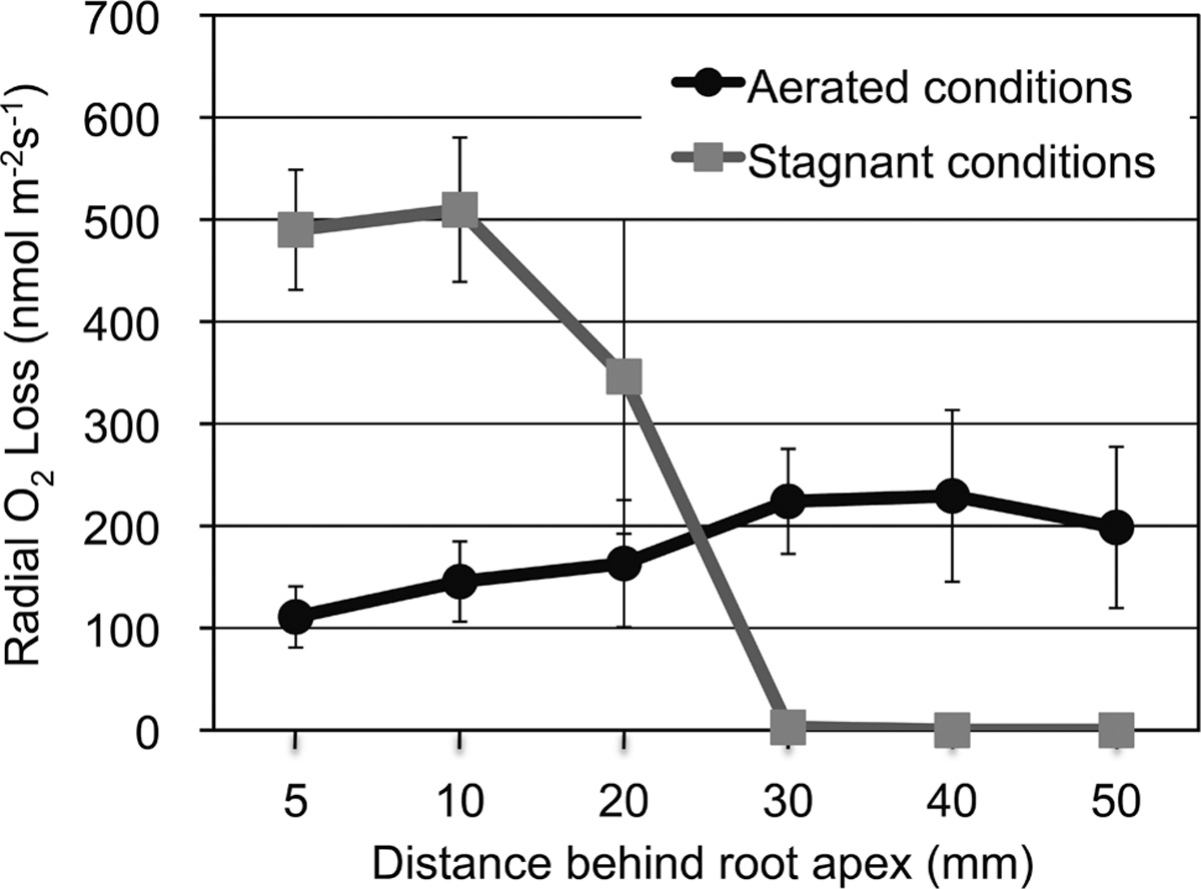
**Rates of ROL along intact adventitious roots of rice grown under aerated or stagnant deoxygenated conditions**. The experiment was conducted following the methods of Colmer et al. (1998) with minor modifications. Rice (cv. Nipponbare) was grown in a 28°C, continually lit growth chamber. Nine-day-old plants were grown in aerated nutrient solution (Aerated conditions) or stagnant deoxygenated agar solution (Stagnant conditions) for 14 or 15 days before measurements were taken along adventitious roots (80-130 mm long). ROL measurements were performed at 27-29°C under light conditions using cylindrical root-sleeving O_2 _electrode. Values are means (*n *= 3) ± SD.

The roots of some wetland species have constitutively present barriers to ROL (*e.g. J. effusus*; Visser et al. 2000), whereas in other species such as rice and *Hordeum marinum *the barrier to ROL is induced during growth under anaerobic conditions (Colmer 2003b; Colmer et al. 1998; Garthwaite et al. 2003; Kotula et al. 2009a; Shiono et al. 2011). Analysis of the spatial patterns of ROL along rice roots has revealed that O_2 _leakage from the basal regions of the long roots under stagnant conditions is quite low (Figure 4), but there are large amounts of O_2 _flux from the root apexes (Figure 4) and numerous short lateral roots that appear near the base of the main axes (Armstrong 1971a; Armstrong et al. 1996, Colmer 2003b). The barrier to ROL, together with reoxidation of the rhizosphere around the root tips and lateral roots, enables elongation of the roots into the anoxic environment and restricts the entry of toxic compounds from highly reduced soils (Armstrong 1979; Armstrong et al. 1996; Colmer and Voesenek 2009).

Despite the importance of the barrier to ROL, there are few data available on the O_2 _permeability coefficient across the cell layers exterior to the aerenchyma. Recently, Kotula and Steudle (2009) developed a gas perfusion technique to measure the O_2 _permeability of the outer cell layers of the roots and applied the technique to rice grown under either aerated or stagnant deoxygenated conditions. Plants grown in the stagnant O_2_-deficient conditions of the external growth medium showed much lower O_2 _permeability than plants grown in an aerated solution. The variation in O_2 _permeability, either by blocking apoplastic pores or killing living tissues, indicated that physical resistance is the dominating factor impeding O_2 _loss from rice roots, although respiratory O_2 _consumption may contribute to low rates of ROL (Kotula et al. 2009b). Strong physical impedance to radial O_2 _diffusion in rice roots has already been shown by Armstrong (1971b). In this study, a barrier to ROL was evident in the adventitious roots of rice, even when respiration was inhibited by cooling the root medium to 3°C. Similar findings have been reported by Armstrong et al. (2000) and Garthwaite et al. (2008) in the roots of *P. australis *and *H. marinum*, respectively. In *H. marinum *the physical barrier appeared to account for 84% of the reduction in O_2 _loss and respiratory activity for 16% (Garthwaite et al. 2008).

#### Anatomical and chemical nature of the barrier to ROL

**Figure 5:**
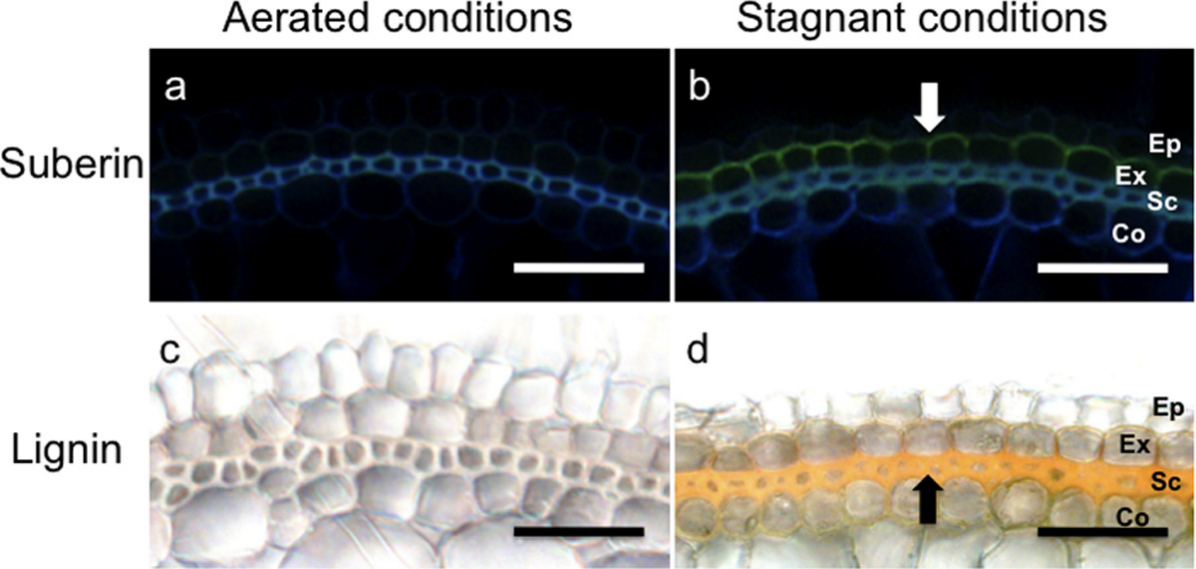
**Staining for suberin and lignin in the outer cell layers of rice roots grown under aerated or stagnant deoxygenated conditions**. Nine-day-old rice plants were grown in aerated nutrient solution (Aerated conditions; a, c) or stagnant deoxygenated agar solution (Stagnant conditions; b, d) for 14 days. Basal parts (10-20 mm regions from the root-shoot junction) of the adventitious roots were sliced into 80-μm-thick sections, and were incubated in lactic acid saturated with chloral hydrate at 70°C for 1 h for clearing. For suberin staining, sections were stained with Fluorol Yellow 088 at room temperature for 1 h and observed under UV-light with epifluorescence microscopy (a, b). For lignin staining, sections were stained for 5 min with phloroglucinol/hydrochloride at room temperature to visualize lignin with cinnamyl aldehyde groups (c, d). White arrow indicates yellow-green fluorescence of suberin at hypodermis/exodermis and black arrow indicates orange-red pigmentation of stained lignin at the sclerenchyma. Ep, epidermis; Ex, exodermis; Sc, sclerenchyma; Co, cortex. Scale bars = 50 μm.

It seems that suberization and/or lignification of the cell walls in the root layers exterior to the aerenchyma is implicated in the development of a tight barrier to ROL (Figure 5; Armstrong et al. 2000; Garthwaite et al. 2008; Kotula et al. 2009a; Soukup et al. 2007). In a recent study in rice, rates of ROL from the roots of plants grown under aerated or deoxygenated conditions were quantified, and the results were combined with parallel histochemistry and analytical chemistry (Kotula et al. 2009a). Deoxygenated conditions induced the early development of Casparian bands and suberin lamellae in the exodermis and of lignin in densely packed, uniseriate sclerenchymal cells located interior to the exodermis (Figure 5). In agreement with the results of the histochemical analyses, quantitative analyses using gas chromatography and mass spectrometry have shown that the levels of suberin (both aromatic and aliphatic domains), as well as lignin, released from the outer root sleeves are several times greater in plants grown in oxygen-deprived media compared with plants grown in aerated solution (Kotula et al. 2009a; Ranathunge et al. 2011). Independent of the growth conditions, the total amounts of suberin and lignin increase along the roots towards the basal zones (Kotula et al. 2009a; Ranathunge et al. 2011). Although these studies have shown a direct relationship between changes in O_2 _permeability and the formation of apoplastic barriers, the precise nature of the ROL barrier remains unclear. Namely, the relative contributions of suberin and lignin in limiting ROL are not well known; potentially, one of the two might not be needed for formation of the barrier (Kotula et al. 2009a). Function of lignin in preventing ROL may not be applied to all plant species. In Amazonian tree species (De Simone et al. 2003), as well as in *P. australis *(Soukup et al. 2007), resistance to ROL is correlated only with the deposition of suberin, but not lignin. Histochemical solute penetration studies using periodic acid have further confirmed that in the roots of *P. australis *the suberized exodermis--not the lignified sclerenchyma, which is easily penetrated by periodic acid--is the radial permeation barrier (Soukup et al. 2007). Shiono et al. (2011) found that suberin increased prior to changes in lignin in rice, suggesting that deposition of suberin is more important for the ROL barrier formation than lignin. However, the suberin and lignin deposits were not evident in the roots within the first 2 days of stagnant treatment, during which time barrier induction was already complete. In long adventitious roots (105-130 mm in length), barrier to ROL was well formed within 24 hours under stagnant deoxygenated conditions and dark granules of high-density material were observed by transmission electron microscopy in the spaces between the exodermal cells and also between the sclerenchyma cells in roots at 48 hours after the stagnant treatment (Shiono et al. 2011). This result suggests that these microstructural changes also contribute to the diminution of ROL in the rice root. The precise nature of the barrier to ROL still needs to be more clearly elucidated (see below).

#### The barrier to ROL vs. nutrient and water uptake

Although the barrier to ROL helps wetland plants to tolerate waterlogging, it may also reduce water and nutrient uptake (Armstrong 1979; Koncalová 1990). For example, the rates of NH_4_^+ ^and NO_3_^- ^net uptake in the basal region of rice roots were about 30% of those in maize, even when the plants were grown in aerated solution (Colmer and Bloom 1998) and the permeability of roots to water is substantially smaller in rice than in maize (Hose et al. 2001; Miyamoto et al. 2001). However, a recent study demonstrated that the early formation of apoplastic barriers in the endodermis and exodermis of rice roots in stagnant solutions does not significantly affect hydraulic conductivity (Ranathunge et al. 2011). This is in agreement with the earlier findings of Garthwaite et al. (2006) in stagnantly grown *H. marinum*, in which induction of the barrier to ROL did not impede the water permeability of the roots. In contrast to water flow, stagnant growth conditions markedly reduce the permeability of the rice roots to oxygen and to ions such as Fe^2+^, Cu^2+^, and NaCl (Armstrong and Armstrong 2005; Kotula et al. 2009b; Krishnamurthy et al. 2009; Ranathunge et al. 2011). The extra suberin and lignin deposited in the roots in stagnant solutions may effectively clog the wall pores, making a barrier sufficient to block the passage of oxygen and ions, but not water, which is mainly bulk and viscous in nature (Kotula et al. 2009b; Ranathunge et al. 2004, 2011). It appears that rice roots living in anaerobic media can retain oxygen in the aerenchyma while taking up sufficient water (Kotula et al. 2009b; Ranathunge et al. 2011). This is achieved because of differences in the transport mechanisms of oxygen and water. When measured with heavy water, the diffusional water permeability of the outer part of the rice root was an order of magnitude smaller than that of oxygen (Ranathunge et al. 2004; Kotula et al. 2009b; Kotula and Steudle 2009). However, diffusional water permeability was smaller than the bulk water permeability by a factor of 600-1400. The latter parameter is the one that is important during water uptake (Steudle and Peterson 1998). This suggests that rice has evolved an optimum balance between water uptake and O_2 _loss. Water moves predominantly through the porous apoplastic pathway by using a hydrostatic pressure gradient (Ranathunge et al. 2004), whereas the movement of O_2 _ought to be appreciable over the whole inter-cell interface and is diffusional in nature (Kotula et al. 2009b; Ranathunge et al. 2011).

#### Formation of the barrier to ROL

There are major uncertainties regarding the signals involved in the formation of an inducible barrier to ROL. Colmer et al. (2006) showed that ethylene, which promotes the induction of lysigenous aerenchyma formation (Justin and Armstrong, 1991), did not induce a tight barrier to ROL in rice roots, indicating that these two root aeration traits, which are considered to act synergistically to enhance O_2 _diffusion to the root apex, appear to be differentially regulated. By contrast, a significant decline in ROL, which is correlated with suberization and lignification of the outer cell layers, is observed after the exposure of rice roots to carboxylic acids (*e.g*. acetic acid, propanoic acid, butyric acid, and caproic acid; Armstrong and Armstrong 2001) and sulfide (in the form of phytotoxins produced by micro-organisms in waterlogged soils; Armstrong and Armstrong 2005). Similar effects are found when carboxylic acids are applied to the roots of *H. marinum *(Kotula, Colmer, and Nakazono, unpublished). However, the reduction in ROL from the roots of rice and *H. marinum *exposed to toxic levels of carboxylic acids and sulfide was associated with injury, rather than with a specific signal for induction of the barrier to ROL (Armstrong and Armstrong 2001; Colmer 2003a).

Recently, Fleck et al. (2011) showed that Si nutrition increased suberization and lignification of rice roots, which was accompanied by silicic acid-triggered transcription of genes associated with suberin and lignin biosynthesis. As a consequence of suberization and lignification of the outer root cell layers, the oxidation power of the rice roots was reduced. Although it is suggested by Fleck et al. (2011) that altered levels of silicic acid play a role in promoting the biosynthesis of suberin and lignin, the signal involved in inducible ROL barrier formation remains unclear.

### Perspectives

Although great progress has been made in our understanding of the mechanisms involved in adaptation to submergence or waterlogging, there are still gaps in our knowledge, mainly in regard to the signaling pathways and molecular processes. The genetic regulation of the formation of lysigenous aerenchyma and the barrier to ROL remains to be determined. Recently, Nakazono and his colleagues (Rajhi et al. 2011; Shiono, Yamazaki, and Nakazono, unpublished) have been investigating the expressions of genes associated with the formation of inducible lysigenous aerenchyma and the barrier to ROL by using laser microdissection-mediated microarray analysis of the cortex in maize roots and the outer cell layers in rice roots, respectively, under anaerobic conditions. This approach should help to identify important genes involved in the formation of these two structures. Further insights into the nature of the barrier to ROL and the molecular mechanism of inducible barrier formation could be achieved from the characterization of suberin or lignin formation-affected rice mutants in comparison with the respective wild types (Ranathunge et al. 2011).

## Conclusions

This review summarizes what is known about the physiological and molecular mechanisms used by rice to cope with submergence and waterlogging. For submergence, the mechanisms include internal aeration and growth controls (*i.e*., a quiescence strategy or an escape strategy). For waterlogging, the mechanisms include formation of aerenchyma and a barrier to ROL. These adaptive traits enable rice plants to have high tolerance to submergence or waterlogging compared with other dryland crops. An advantage of rice is that its genome has been fully sequenced and many tools for studying its molecular biology and genetics (*e.g*. oligo microarrays, mutant collections, and databases) have been developed. These resources should accelerate our understanding of the mechanisms involved in adaptation of rice to excess water stress and should lead to their introduction into dryland crops.

## Competing interests

The authors declare that they have no competing interests.

## Authors' contributions

All of the authors contributed equally to the drafting and revising of this paper and have read and approved the final manuscript.
